# The awesome lysosome

**DOI:** 10.15252/emmm.201505966

**Published:** 2016-01-19

**Authors:** Andrea Ballabio

**Affiliations:** ^1^ Telethon Institute of Genetics and Medicine (TIGEM)NaplesItaly; ^2^ Medical GeneticsDepartment of Translational MedicineFederico II UniversityNaplesItaly; ^3^ Department of Molecular and Human GeneticsBaylor College of MedicineHoustonTXUSA; ^4^ Jan and Dan Duncan Neurological Research InstituteTexas Children HospitalHoustonTXUSA

**Keywords:** Genetics, Gene Therapy & Genetic Disease

## Abstract

In the early 50s, Christian De Duve identified a new cellular structure, the lysosome, defined as the cell's “suicide bag” (de Duve, 2005). Sixty years later, it is clear that the lysosome greatly exceeded the expectations of its discoverer. Over 50 different types of lysosomal storage diseases have been identified, each due to the deficiency or malfunction of a specific lysosomal protein. In addition, an important role of the lysosome has been unveiled in several common human diseases, such as cancer, obesity, neurodegenerative diseases, and infection. Recent studies have led to the identification of a lysosome‐to‐nucleus signaling pathway and a lysosomal gene network that regulate cellular clearance and energy metabolism. These observations have opened a completely new field of research and changed our traditional view of the lysosome from a dead‐end organelle to a control center of cell metabolism. An important challenge for the future will be to exploit these discoveries to identify modulators of lysosomal function that may be used to treat human diseases.

## The lysosome at a glance

Cellular organelles enable the spatial clustering of molecules, thus favoring their interactions in microenvironments ideally suited for specific complex functions. A well‐known function of the lysosome is to degrade and recycle cellular waste. Extracellular materials reach the lysosome mainly through endocytosis and phagocytosis, while a completely different process, autophagy, mediates the delivery of intracellular materials. Autophagy is activated by a broad range of cellular stress‐inducing conditions and mediates the degradation of protein aggregates, oxidized lipids, damaged organelles, and intracellular pathogens. The process typically involves the formation of double membrane‐bound vesicles, the autophagosomes, which sequester cytoplasmic material and then fuse with lysosomes. Materials that reach the lysosome are degraded by lysosomal hydrolases, and the resulting breakdown products are used to generate new cellular components and energy in response to the nutritional needs of the cell. Lysosomes are also involved in an “unconventional” secretory pathway known as lysosomal exocytosis, which plays an important role in various physiological processes such as plasma membrane repair, immune response, and bone resorption.

The lysosomal lumen has an acidic pH close to 4.5 and contains approximately 60 different soluble hydrolytic enzymes, which are directly involved in the degradation of metabolites. The lysosomal membrane contains proteins such as transporters, ion channels and SNAREs that mediate different aspects of lysosomal function, as well as the vATPase complex that mediates lysosomal acidification. In addition to lumenal and integral membrane proteins, an expanding number of proteins and of protein complexes have been found to associate, under particular conditions, with the lysosomal surface. The activity of such proteins may be either directly influenced by the lysosome, or in turn may regulate or mediate specific aspects of lysosomal function. Notable examples are the mTORC1 kinase complex, whose activity depends on lysosomal nutrient content (Zoncu *et al*, 2011), and the BORC protein complex, which regulates lysosome positioning (Pu *et al*, 2015). We know, however, the precise function of only a small fraction of the known lysosomal proteins, and it is likely that additional ones remain to be identified.

## The expanding role of the lysosome in human diseases

Mutations in genes encoding proteins involved in lysosomal function cause lysosomal storage diseases (LSDs), a group of about 50 inherited disorders characterized by the progressive accumulation of undegraded substrates inside the lysosome. Patients with LSDs present with a debilitating, multi‐systemic phenotype often associated with early‐onset neurodegeneration. Unfortunately, therapeutic options are still inefficient or simply unavailable for most LSDs. How exactly the storage of undegraded material in LSDs causes cellular and tissue dysfunction and clinical symptoms has yet to be fully elucidated. A common consequence of pathological lysosomal storage is an impairment of autophagy, which leads to the secondary accumulation autophagy substrates.

Notably, the lysosome has also been found to be crucially involved in a variety of common disease conditions, such as neurodegenerative diseases, infection, obesity, and cancer. Induction of the lysosomal–autophagic pathway was detected in pancreatic cancer (Perera *et al*, 2015). Furthermore, cancer cells were found to be more prone to lysosomal membrane permeabilization (Aits & Jaattela, 2013). In recent years, lysosomal gene mutations have been identified in an increasing number of patients with common neurodegenerative diseases, such as Parkinson's and Alzheimer's. For example, heterozygosity for mutations in the gene encoding glucocerebrosidase predisposes to Parkinson's disease (Sidransky *et al*, 2009) via a mechanism that is still unclear. Interestingly, homozygosity for mutations in the same gene causes Gaucher disease, the most common neurodegenerative LSD.

Additionally, aggregate‐prone proteins such as huntingtin and α‐synuclein that are involved in Huntington's and Parkinson's diseases, respectively, are degraded by the lysosomal–autophagic pathway and can be eliminated by inducing autophagy. These findings emphasize the importance of the lysosomal–autophagic pathway in neurodegenerative diseases and that therapeutic strategies aimed at rescuing and/or enhancing this pathway may have a broad impact on human health.

## TFEB and the CLEAR network regulate lysosomal biogenesis

How the cell controls lysosomal function has remained unanswered and unexplored for a long time. A systems biology approach led to the discovery that lysosomal biogenesis and autophagy are transcriptionally regulated by a gene network, named CLEAR, and by its master gene TFEB (Sardiello *et al*, 2009; Settembre *et al*, 2011), a member of the helix‐loop‐helix (HLH) leucine zipper family of transcription factors, providing the first evidence that lysosomal function is globally regulated and how this might occur.

**Figure 1:**
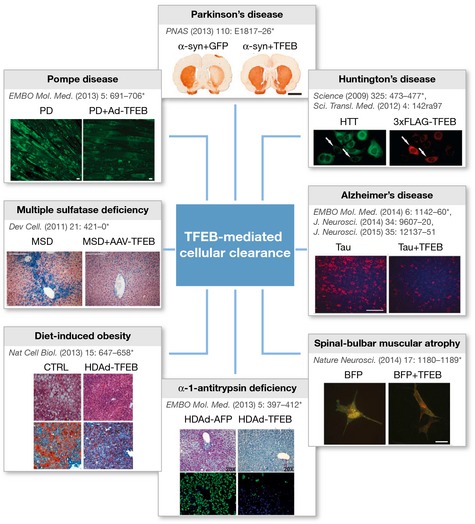
TFEB‐mediated cellular clearance TFEB overexpression promotes cellular clearance in mouse models of human diseases. Representative figures from published studies demonstrating clearance of accumulating substrates in LSDs, Parkinson's, Alzheimer's, and Huntington's diseases, α1‐anti‐trypsin deficiency, and spinal bulbar muscular atrophy. The asterisks indicate the papers in which the original images were published.

We now know that TFEB plays a fundamental role in cell homeostasis and provides us with an unprecedented tool to globally induce lysosomal function and autophagy *in vitro*, as well as *in vivo*. Figure 1 shows the disease models in which overexpression of TFEB effectively reduces the amount of accumulated substrates via the induction of the lysosomal–autophagic pathway. These include LSDs but also α1‐anti‐trypsin deficiency and spinal bulbar muscular atrophy, as well as common diseases such as Parkinson's, Alzheimer's, and Huntington's. In all cases TFEB overexpression resulted in a significant rescue of the disease phenotype (Settembre *et al*, 2013). Recent studies showed that another HLH transcription factor highly homologous to TFEB, TFE3, regulates a similar set of genes and is able to promote cellular clearance (Martina *et al*, 2014). The possibility of globally modulating lysosomal function by acting on TFEB/TFE3 and on the CLEAR network may lead to a novel therapeutic strategy with potential applicability to many diseases. However, potential side‐effects of such approach will have to be carefully evaluated.

## The lysosome as a signaling hub

The kinase complex mTORC1 (mechanistic target of rapamycin complex 1), a master controller of cell and organism growth, was recently shown to exert its activity on the lysosomal surface and to become inactive when released from the lysosome (Zoncu *et al*, 2011). mTORC1 belongs to a complex signaling machinery that responds to the lysosomal amino acid content via a nutrient sensing mechanism. Thus, the lysosome plays an important and unexpected role in cell signaling by controlling the activity of mTORC1.

Interestingly, mTORC1 regulates TFEB subcellular localization and activity. mTORC1‐mediated phosphorylation of TFEB specific serine residues, which occurs on the lysosomal surface (Martina *et al*, 2012; Roczniak‐Ferguson *et al*, 2012; Settembre *et al*, 2012), keeps TFEB inactive in the cytoplasm. Under nutrient‐rich conditions, active mTORC1 promotes biosynthetic pathways and blocks autophagy. A variety of stimuli, such as starvation and lysosomal stress, inhibit mTORC1 activity, thus inducing TFEB nuclear translocation and promoting lysosomal biogenesis and autophagy.

**Figure 2:**
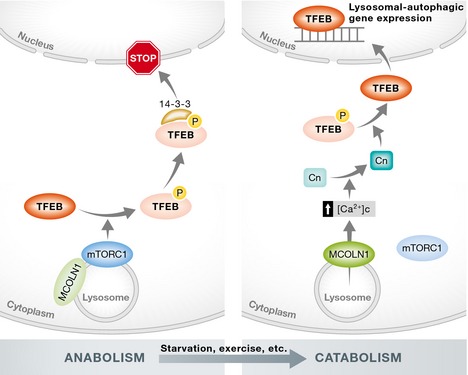
A lysosome‐to‐nucleus signaling mechanism Signaling mechanisms that regulate TFEB nuclear translocation. Under normal feeding conditions, TFEB is phosphorylated by mTORC1 on the lysosomal surface and is sequestered in the cytoplasm by 14‐3‐3 proteins. During starvation and physical exercise mTORC1 is inactivated and Ca^2+^ is released from the lysosome through MCOLN1. This leads to local calcineurin activation and TFEB dephosphorylation. Dephosphorylated TFEB is no longer able to bind 14‐3‐3 proteins and can freely translocate to the nucleus where it transcriptionally activates the lysosomal/autophagic pathway [modified from (Medina *et al*, 2015)].

In addition, under energy demanding conditions, such as starvation and physical exercise, lysosomal calcium release via the lysosomal calcium channel mucolipin‐1 (MCOLN1) activates the calcineurin phosphatase, which in turn dephosphorylates TFEB, thus promoting its nuclear translocation and activation (Medina *et al*, 2015). Therefore, a lysosome‐to‐nucleus signaling mechanism enables lysosomal function to respond to environmental cues and regulates the switch between cellular biosynthetic and catabolic pathways (Settembre *et al*, 2012). These studies indicate that the lysosome acts as a signaling hub that controls cell metabolism and homeostasis (Fig 2).

## The “lysosomics” approach

After 60 years from its discovery, the lysosome has yet to reveal all its secrets. Future studies may lead to the discovery of additional lysosomal regulatory networks operating at the transcriptional, posttranscriptional (e.g. microRNAs), or posttranslational levels. An example of a lysosomal posttranslational regulatory pathway is the regulation of sulfatases by the sulfatase modifying factor 1 (SUMF1).

Lysosomal function may also be mediated by protein networks via protein–protein interactions. A few examples of such complexes have already been identified, such as the GLB1‐NE1‐GALNS‐CTSA complex in the lysosomal lumen and the above‐mentioned vATPase complex on the lysosomal membrane. Proteomic studies applied to the lysosome may reveal additional examples of such protein complexes and their function.

Another important goal is to further elucidate the signaling pathways that control lysosomal function as well as the role of the lysosome as a signaling hub. The study of these pathways may lead to the discovery of tools able to modulate lysosomal function in a sensitive and selective fashion. Functional genomic approaches (e.g. siRNA‐based) and drug screenings, combined with high content cell‐based assays, will play an important role in this endeavor.

Finally, we need to further define the role of lysosomal mutations as predisposing factors for human diseases. Whole genome, exome, and targeted sequencing of lysosomal genes, combined with functional analysis, will allow to identify new lysosomal genes playing a major role in the pathogenesis of a broad variety of human diseases. The Lysoplex tool (Di Fruscio *et al*, 2015) represents an example of a lysosome‐targeted sequencing platform, which may be used to find both disease‐causing mutations in LSDs and predisposing mutations in common diseases.

In conclusion, the biology and pathophysiology of the lysosome is still far from being completely understood. The integration of “omics” technologies to study the lysosome, an approach that may be termed “lysosomics”, will reveal the full potential of the awesome lysosome.
